# Robust predictors for drug response of patients with acute myeloid leukemia

**DOI:** 10.1371/journal.pone.0343422

**Published:** 2026-02-23

**Authors:** Bahar Tercan

**Affiliations:** Institute for Systems Biology, Seattle, Washington, United States of America; European Institute of Oncology, ITALY

## Abstract

The significant heterogeneity in treatment responses among patients with acute myeloid leukemia (AML) underscores the critical need for accurate drug response prediction. We developed k-Top Scoring Pairs (kTSP) classifiers, ensemble methods that aggregate the relative expression of gene pairs. We compared their accuracy with that of state-of-the-art machine learning methods, linear and radial basis function support vector machines, random forest and elastic net regression classifiers for drug response prediction of patients with AML. Our results demonstrate that kTSP particularly outperforms other methods when the number of sensitive and resistant patients is imbalanced, a common challenge in clinical studies. Our approach is inherently robust to batch effects and uniquely suited for single-patient classification due to its rank-based methodology.

## Introduction

Acute myeloid leukemia (AML) is an aggressive hematological cancer characterized by the clonal accumulation of abnormally differentiated, immature myeloid cells (blasts) in the bone marrow and blood, leading to bone marrow failure [1,2]. The 5-year survival rate for AML is 32.9% [3]. Improved treatment options have enabled AML to be cured in 35%–40% of patients younger and 5% – 15% of patients older than 60 years old [1].

Due to high heterogeneity in AML patients and varied treatment responses, molecular data in AML has predominantly been used for predicting prognosis and deciding on post-remission treatment. While genomic profiling is essential for guiding initial therapeutic decisions in certain subsets, notably the use of FLT3 inhibitors (midostaurin/quizartinib) for FLT3-mutated AML [4,5], and Gemtuzumab Ozogamicin (GO) for *CD33*-positive core-binding factor AML [6,7] — its integration is still limited compared to later stages of therapy. The standard frontline treatment for AML patients who are fit for intensive chemotherapy remains a cytarabine-based (7 + 3) regimen. Furthermore, although the approval of other targeted therapies has been robust, their frontline use as monotherapy or non-intensive combinations (e.g., Venetoclax, IDH inhibitors) is largely restricted to elderly or unfit patients [8].

The Beat AML clinical trial (NCT03013998) provided cytogenetic and mutational data within seven days of sample receipt, allowing for rapid treatment selection. Patients were then assigned to sub-studies based on the dominant clone of their tumors. This molecularly-guided approach resulted in a median overall survival of 12.8 months, significantly longer than the 3.9 months observed in patients treated with the standard of care (induction with cytarabine and daunorubicin [7 + 3 or equivalent] or a hypomethylation agent) [9]. This study demonstrates the importance of precision medicine and paves the way for personalized therapy by providing molecular, clinical, and ex vivo drug response data for individual acute myeloid leukemia (AML) patients.

In this study, we utilize the comprehensive Beat AML dataset [10,11], a multi-phase resource spanning ten years. This dataset includes ex vivo drug sensitivity, clinical annotations, and rich molecular data from hundreds of patients with heterogeneous AML subtypes, providing an ideal foundation for developing drug response predictors.

Previous data-driven studies have used the multi omics data in Beat AML, including selected clinical features, mutations, and gene expression [12,13] to predict patient-specific drug responses. Other studies have used derived attributes from gene expression data, such as cell-type deconvolution [14] and BCL2 signatures [15]. One study is focused on predicting response to particular drugs such as BET inhibitors [16]. Proteomics data from Beat AML patients has been generated and used in drug response classifiers by Pino et *al.* [17] and Gosline et *al.* [18].

We employed a robust machine learning approach, the k-Top Scoring Pairs (kTSP) classifier [19], to predict the *ex vivo* drug response of patient-derived samples from the Beat AML cohort. This method is uniquely suited for clinical translation and is robust to technical variability (batch effects) as it relies solely on the relative expression of a few informative gene pairs. We benchmarked the predictive accuracy of kTSP against state-of-the-art models, including random forest, linear and radial basis function (RBF) support vector machines (SVM) and elastic net regression. Another uniqueness of our study is that it is the first ex vivo drug response prediction work on the venetoclax-containing combination drug response data obtained from Eide et *al.* [20]. We applied our classifiers on an external AML cohort, Functional Precision Medicine Tumor Board (FPMTB) [21] to evaluate the generalizability of them. We also reported the consistency of rules across different temporal (Beat AML Waves *1 + 2* and Waves *3 + 4) and clinical cohorts de novo or not*.

Crucially, our completely data-driven approach is inherently robust: it does not require batch correction or data normalization. This property allows for single-sample classification, enabling clinicians and researchers to classify individual samples simply by checking the majority vote across the classifier gene pairs—a significant practical advantage in a clinical setting. We provide the classifier gene pairs for both single drugs and venetoclax-containing combination drugs as supplementary files.

## Materials and methods

### Data acquisition

In this study we used the Beat AML dataset [10,11] which was collected over ten years in two phases: waves *1 + 2* and waves *3 + 4*. It contains ex vivo drug sensitivity, clinical annotations, and RNA (Agilent SureSelect Strand-Specific RNA Library Preparation Kit on the Bravo robot (Agilent)) and DNA (Illumina Nextera RapidCapture Exome capture probes and protocol) sequencing data from patients with *de novo*, *transformed*, and *therapy-related* AML, as well as patients with *relapse*. De novo patients are the AML patients with no prior history of Myelodysplastic Syndromes (MDS), Myeloproliferative Neoplasms (MPN), or chemotherapy. Transformed AML evolves from a prior myeloid disorder (MDS or MPN), and therapy related AML occurs after exposure to chemotherapy or radiation for a different disease. Relapse is the recurrence of disease after achieving remission.

To analyze the generalizability of our relative gene expression-based kTSP classifiers, we applied them to an independent cohort of Acute Myeloid Leukemia (AML) patients from the FPMTB [21], which provided functional, genomic, and transcriptomic data.

All the data we used in this is publicly available and was downloaded on July 10, 2024. We did not have access to information that could identify individual participants during or after data collection.

### Gene expression normalization

We converted the RPKM normalized gene expression to transcript per million (TPM) for the Beat AML dataset with the Equation 1 [22].


$$TPM_{i}=\left(\frac{{RPKM}_{i}}{\sum_{j}^{N}{RPKM}_{j}}\right)\times {\text{1}\text{0}}^{\text{6}}$$ (1)

where *i* is the index of a gene and *N* is the number of genes in a sample.

We converted the gene counts from FPMTB cohorts into TPM using the convertCounts function from DGEobj.utils R library (version 1.0.6). We kept only protein-coding genes and used log2 -normalized TPM matrices in the classification tasks.

### k-Top scoring pairs classifier

For classification, we utilized the kTSP algorithm [19], an ensemble extension of the Top Scoring Pair (TSP) algorithm [23]. The TSP algorithm operates as a simple binary “rule” based on the relative expression of two genes (geneA≶geneB). The kTSP classifier aggregates these rules from distinct TSPs, making final predictions through unweighted majority voting (the default setting). This rank-based approach is interpretable, robust to batch effects, and invariant to any monotonic transformation of the data. We implemented kTSP using the Bioconductor switchBox package (version 1.45.0) [24,25]. The optimal number of pairs, *k*, was selected using the analysis of variance approach [26] during the training phase, ensuring no data leakage from the test data set. Given our aim to maintain a simple and clinically translatable classifier, we restricted the search range for *k* to *1*–*15* gene pairs for all single-drug and combination-drug response predictions.

### Drug sensitivity quantitation and thresholding

In the Beat AML ex vivo drug response profiling, drug response was quantified using probit-modeled Area Under the Curve (AUC) values (theoretical range 0–300). For each compound, a seven-point concentration series (typically 10 μM to 0.0137 μM) was log10-transformed, and a probit regression curve was fitted to the viability data using maximum-likelihood estimation for slope and intercept [11,20,27]. The AUC was normalized such that a value of 100 represents a non-responsive profile (no change in viability relative to controls). Consequently, AUC < 100 was defined as ‘Sensitive,’ indicating a drug-induced reduction in cell viability, otherwise the sensitivity call was defined as ‘Resistant.’ Values exceeding 100 means the cells actually grew more than the control, the curve fit shifted upward possibly due to experimental noise or drug enhanced growth. This thresholding strategy is consistent with established Beat AML standards [17,18] and reflects the inverse correlation between AUC and drug potency.

For the external cohort analysis, we utilized the Selective Drug Sensitivity Scores (sDSS) from the FPMTB dataset. Selective drug-sensitivity scoring enables normalization of the individual patient’s responses against normal cell responses [28,29]. DSS is a metric dependent on the AUC. It is effectively a normalized version of AUC where $C_{max}$ and $C_{min}$ are the maximum and minimum concentrations at which the drug was screened. The AUC over the dose range $\left(x_{\text{2}}-x_{\text{1}}\right)$ where the responses exceed a user-specified minimum activity level ($A_{min}$) can be calculated either using analytical or numerical integration. Equation 2 shows the formula for ${DSS}_{\text{1}}$


$${DSS}_{\text{1}}=\frac{AUC\;-\;A_{min}\left(x_{\text{2}}-x_{\text{1}}\right)}{\left(\text{1}\text{0}\text{0}\;-A_{min}\right)\left(C_{max}-C_{min}\right)\;}$$ (2)

To penalize the compounds that are effective at higher tested concentrations only, the ${DSS}_{\text{1}}$ summary score is further normalized by the logarithm of the top asymptote ($R_{max}$) of the estimated dose-response model that corresponds to the maximal estimated response of the drug (Equation 3):


$${DSS}_{\text{2}}=\frac{{DSS}_{\text{1}}}{{log}_{\text{1}\text{0}}R_{max}}$$ (3)

To prioritize drugs that demonstrate efficacy across a broad therapeutic window, rather than those active only at the highest concentrations, the scoring algorithm was refined (Equation 4):


$${DSS}_{\text{3}}={DSS}_{\text{2}}\frac{x_{\text{2}}-x_{\text{1}}}{C_{max}-C_{min}}$$ (4)

With each version of the DSS-score, the selective drug sensitivity score (sDSS) is calculated by subtracting the average of the control DSSs from the patient DSS (Equation 5).


$$sDSS={DSS}_{p}-mean\left({DSS}_{c}\right)$$ (5)

### Benchmarking classifiers

To benchmark the performance of the kTSP approach, we employed several state-of-the-art machine learning models: SVM with linear and RBF kernels [30–32], random forest (an ensemble tree-based approach) [33], and elastic net regression (which combines Lasso and Ridge regularizations) [34]. SVMs utilize the kernel trick to transform nonlinearly separable data into a linearly separable space. For all classifiers outside of kTSP, model training and tuning were performed using the caret R package (version 7.0.1).

### Statistical analysis and model evaluation

For the SVM, random forest, and elastic net regression classifiers, we optimized parameters exclusively on the Beat AML Waves *1 + 2* training data using a 5-fold cross-validation (CV) scheme with the caret R package’s random search method. For SVM and elastic net, z-score normalization was applied to the training data. Crucially, no data from the test dataset (Beat AML Waves *3 + 4*) was used in this parameter search.

To address the common challenge of class imbalance (sensitive vs. resistant samples), we employed the Synthetic Minority Over-sampling Technique (SMOTE) [35] data sampling option during the random search process.

Model performance was assessed using several metrics: balanced accuracy (the arithmetic mean of sensitivity and specificity), the Area Under the ROC Curve (AUROC), sensitivity, and specificity. All analyses were conducted using the R programming language (version *4.5.0*).

For external cohort validation, we trained the classifiers on Beat AML 1–4 cohorts and applied them to the FPMTB cohort. The 95th percentile of the sDSS distribution of all drugs measured for all samples was used as a threshold for sensitivity calling for the FPMTB cohort as it is suggested in the paper where the FPMTB dataset was provided.

S2 Fig shows the flowchart of the analyses performed in this manuscript.

## Results

### Predicting drug responses of Beat AML samples

In our first analysis, we trained our models, kTSP and comparator state of the art machine learning models using the Beat AML waves 1 + 2 cohort and tested them on the Beat AML waves 3 + 4 cohort. To ensure robust training and validation of the models, we filtered the drug response data to include only those agents with sufficient sample size. Specifically, only drugs with at least 20 sensitive and 20 resistant samples in the training set (Beat AML Waves *1 + 2)* and at least 10 sensitive and 10 resistant samples in the test set (Beat AML Waves *3 + 4*) were retained for analysis. This approach was applied separately for single drugs and venetoclax-containing combination drugs.

**Fig 1:**
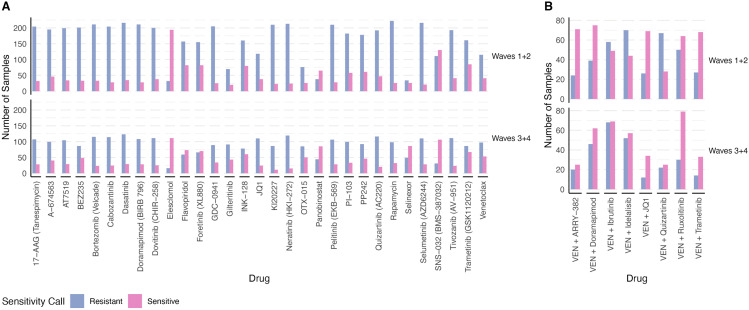
Response to single and venetoclax containing combination drugs. **(A)** The number of sensitive and resistant samples for each single drug in Beat AML Waves 1 + 2 and Waves 3 + 4 cohorts for single drugs (**B)** and for venetoclax containing combination drugs (for combination drug response prediction, drug response from Eide et al [20] and gene expression from Beat AML dataset were used). Area Under Drug Response Curve (AUC)=100 was used as a cut off for sensitivity calling for both single drugs and drug combinations. Note: Only drugs with at least 20 sensitive and 20 resistant samples in the training set (Waves 1 + 2) and at least 10 sensitive and 10 resistant samples in the testing set (Waves 3 + 4) were included in the analysis.

Fig 1 illustrates the distribution of sample counts for both the training and testing cohorts across the included single drugs and combinations.

**Fig 2:**
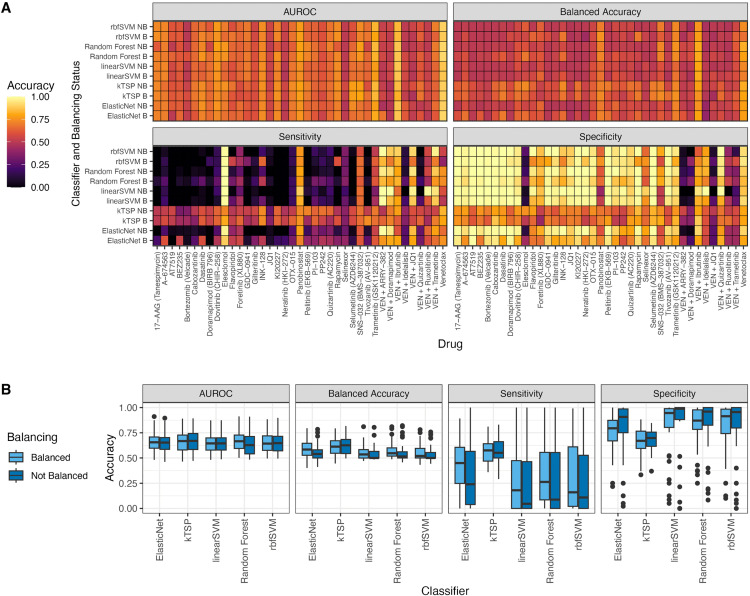
Comparative accuracy of kTSP, random forest, linear and RBF kernel SVM and elastic net regression classifiers in terms of sensitivity, specificity, balanced accuracy and Area Under the ROC Curve (AUROC) for single and venetoclax containing combination drug response prediction when the training data is balanced (with SMOTE method) and not balanced (A) For individual drug (B stands for Balanced and NB stands for Not Balanced in y-axis). (**B)** All drugs together.

We compared the predictive accuracy of kTSP against SVM (linear and RBF kernels), random forest, and elastic net regression (Fig 2). We also examined the effect of addressing class imbalance using the Synthetic Minority Over-sampling Technique (SMOTE) during training. In terms of AUROC, balancing the training data did not improve results for any algorithm. However, all comparator algorithms (excluding kTSP) benefited from balancing in terms of balanced accuracy, suggesting improved success in classifying the minority class. The minority-class refers to a class within a dataset that contains significantly fewer instances compared to other classes.

Fig 2 details the comparative performance of all classifiers for individual drugs and performance of classifiers for all drugs together, respectively.

The kTSP algorithm consistently outperformed the other classifiers based on balanced accuracy and accuracy within the minority class. This superiority was evident across single-drug responses (where sensitive samples were typically the minority) and combination responses (where resistant samples were sometimes the minority). This high performance (Fig 2) was achieved despite kTSP’s inherent simplicity, using an average of 10.57 ± 3.38 rules for single drugs and 10.5 ± 3.43 rules for combination drugs (based on at most 30 genes).

Overall, the high prevalence of imbalanced drug responses in AML samples led to the failure of other classifiers to correctly classify the minority class, even after applying SMOTE. The ability of kTSP to maintain high balanced accuracy in the face of imbalance highlights its utility for real-world clinical prediction.

### Testing generalizability on an external cohort, FPMTB

**Fig 3:**
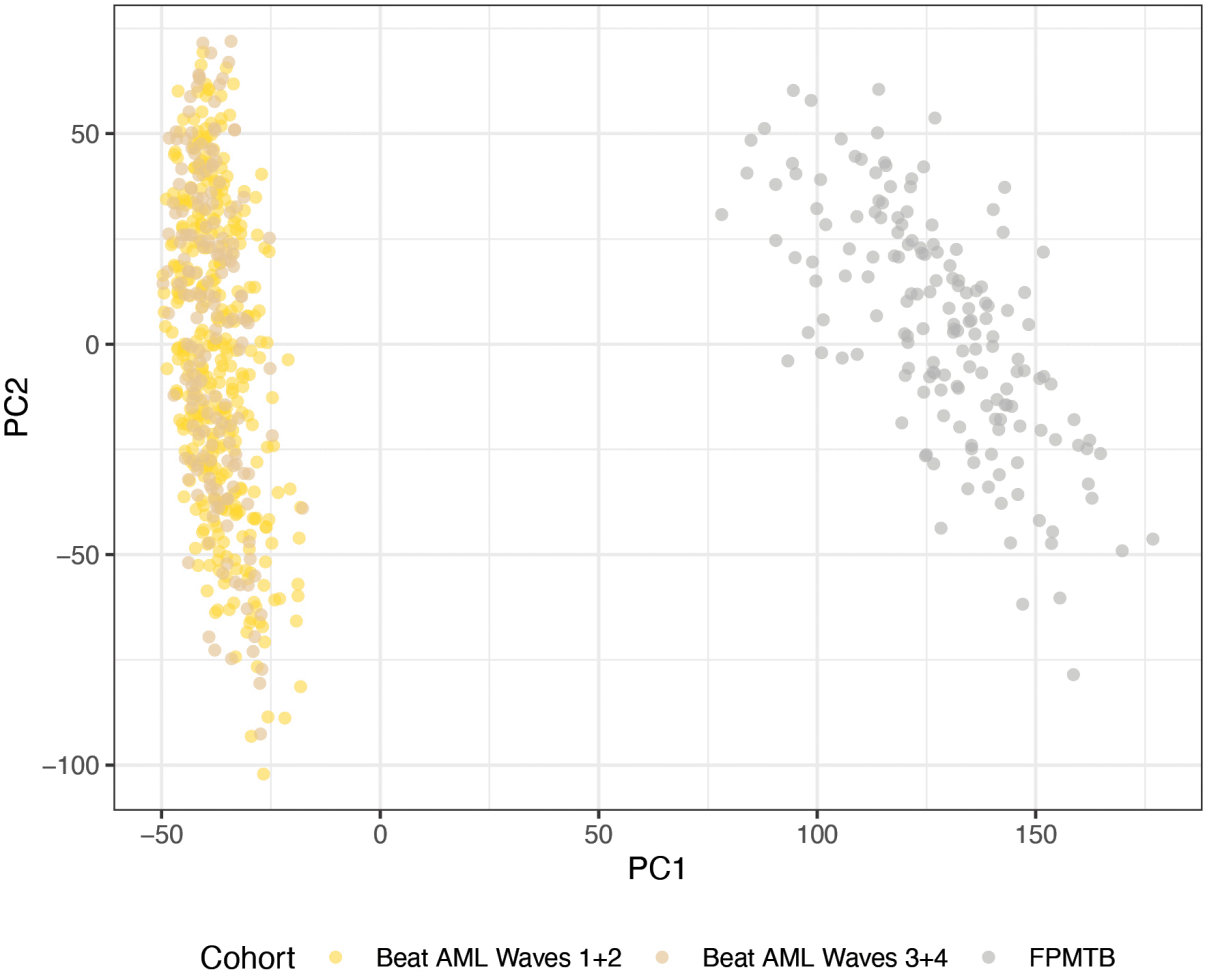
Visualization of the three cohorts, Beat AML waves *1* *+* *2*, Beat AML waves *3* *+* *4* and Functional Precision Medicine Tumor Board (FPMTB), based on the first two principal components. Beat AML dataset, Waves 1 + 2 (371 samples), Waves 3 + 4 (184 samples) has been processed with the same library, SureSelect Strand-Specific RNA Library (Agilent), while the FPMTB (163 samples) dataset which have been processed with different libraries, i.e., NextEra and Scriptseq.

We validated our findings on an independent external cohort, FPMTB. To assess potential batch effects and cohort similarity, we visualized all three datasets (Beat AML Waves *1 + 2*, Waves *3 + 4*, and FPMTB) based on the first two principal components (Fig 3).

Fig 3 shows that the first principal component clearly separates Beat AML cohorts and FPMTB cohort and it indicates that there is a strong batch effect between the two studies which overpowers the biological similarities. In addition to that the FPMTB cluster is more loosely dispersed, suggesting higher variability or heterogeneity which could be caused by different libraries that were used in the gene expression data generation within FPMTB cohort, i.e., NextEra, ScriptSeq compared to the Beat AML group which was processed in the same library, SureSelect Strand-Specific RNA Library (Agilent). This underscores the necessity of rank-based approaches that bypass the need for cross-platform normalization.

**Fig 4:**
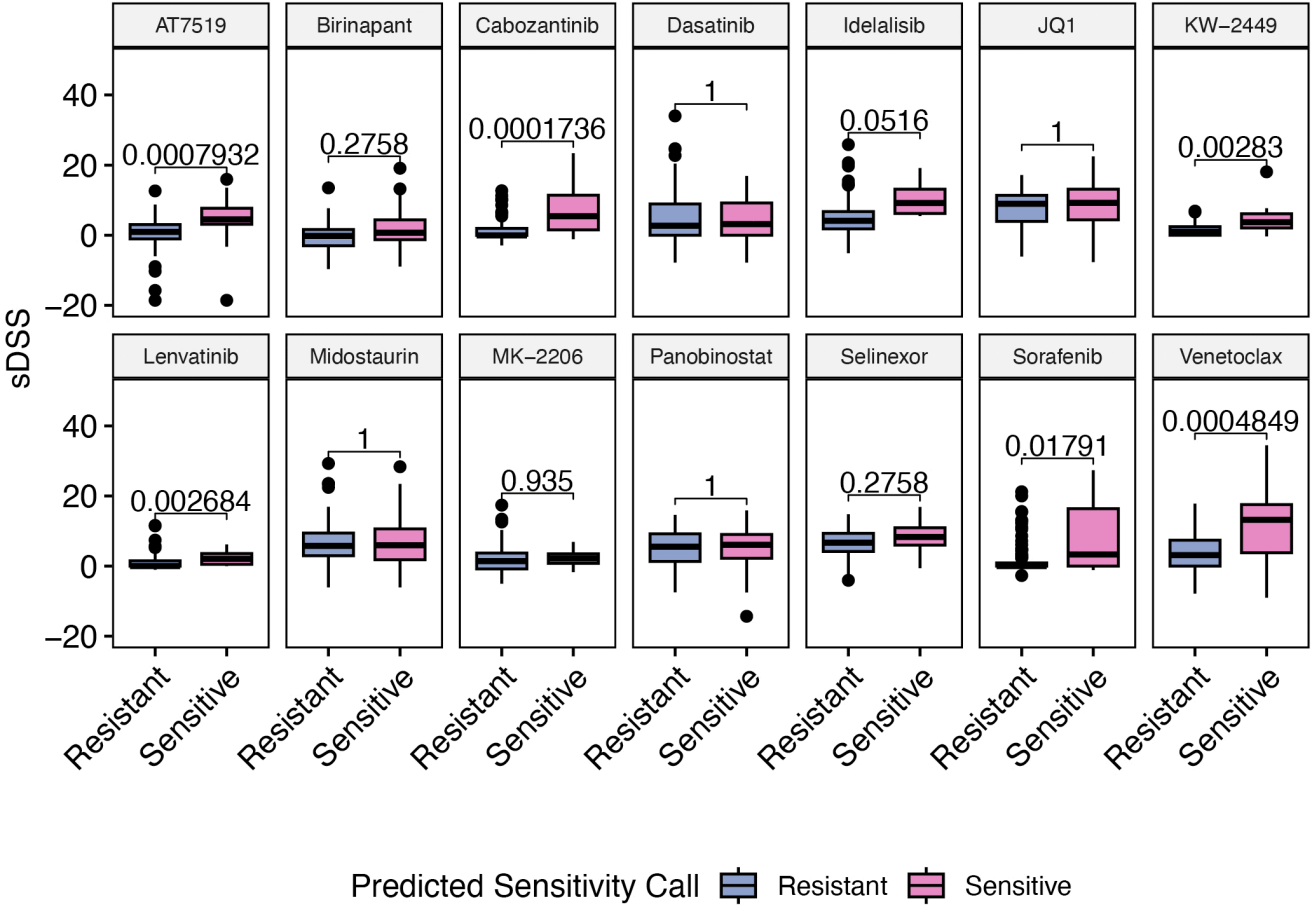
Selective drug sensitivity scores (sDSSs) of the FPMTB validation samples that are predicted to be resistant and sensitive by the kTSP classifier. P values were computed with one sided Wilcoxon test followed by Benjamini-Hochberg multiple test correction. The analysis includes only those drugs for which the kTSP classifier was trained on at least 20 sensitive and 20 resistant samples in the Beat AML dataset.

We observed in the independent FPMTB validation cohort that the selective drug sensitivity scores (sDSS) of samples predicted by kTSP to be sensitive were generally higher than those predicted to be resistant. Specifically, this difference reached statistical significance (P < 0.05) for 6 out of the 14 tested compounds, including Venetoclax and Sorafenib. Fig 4 shows the sDSS values for the samples grouped by their kTSP-predicted sensitivity calls (high sDSS indicates sensitivity to a drug while high AUC suggests resistance).

This significant separation validates the ability of the kTSP classifiers to accurately distinguish patient drug sensitivity in an independent cohort using a distinct (though directionally inverse) drug sensitivity metric, sDSS. The robust performance on this external dataset confirms the strong generalizability of the relative gene expression-based kTSP approach across different AML cohorts.

**Fig 5:**
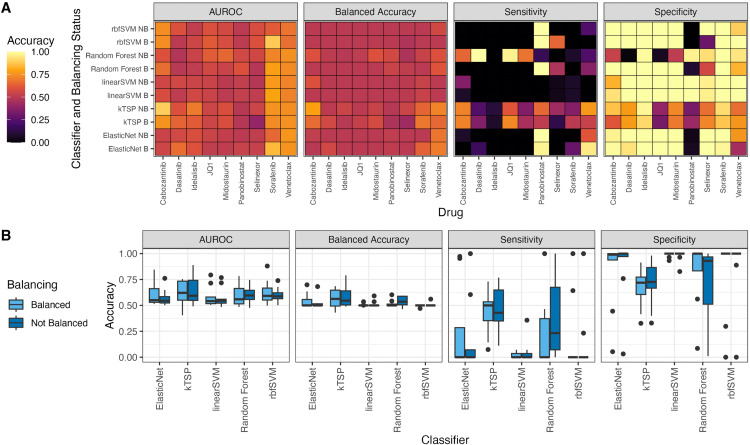
The comparative accuracy of kTSP and other classifiers on the independent FPMTB validation cohort. The drugs that are common to BeatAML and FPMTB, had at least 20 sensitive and 20 resistant samples in Beat AML 1-4 and at least 10 sensitive and 10 resistant samples in the FPMTB cohort.

We also examined the classification accuracy of kTSP and the other classifiers on the FPMTB cohort. To compare the accuracy of kTSP with the state of art classifiers, we repeated the accuracy analysis by training the models on entire BeatAML data and testing on the FPMTB cohort (Fig 5). We included the drugs that are common to BeatAML and FPMTB, which has at least 20 sensitive and 20 resistant samples in Beat AML 1–4 and at least 10 sensitive and 10 resistant samples in the FPMTB cohort. The 95th percentile of the sDSS distribution of all drugs measured for all samples was used as a threshold for sensitivity call for the FPMTB cohort as it is suggested by the paper we got the FPMTB dataset from [21].

The results on the external FPMTB cohort were consistent with the accuracy analysis performed on the Beat AML cohorts. The state of the art classifiers tended to assign all samples to one of the classes in most cases.

### The most predictive genes

From the drug response classifiers that were trained on the Beat AML 1–4 cohort with at least 20 sensitive and 20 resistant samples (Tables in S1, S2 Tables), 83.5% of classifier genes happen to exist in only one of the classifiers, mostly because of the parsimonious nature of feature sets of kTSP classifiers. The most recurrent genes in different classifiers could be considered the ones that are most strongly associated with in vitro drug response. There are two genes that exist in 9 out of 74 classifiers. These genes are leukocyte immunoglobulin-like receptor, *LILRB1* and membrane-associated ring finger (C3HC4) 1), *MARCH1*. There are two genes which exist in 8 of the classifiers,cysteinyl leukotriene receptor 2, *CYSLTR2* and poly(rC) binding protein 3, *PCBP3*. Then followed by 5 genes that existed in 6 classifiers. Focusing on these 4 genes, *LILRB1*’s overexpression is correlated with sensitivity to 8 drugs and its underexpression is correlated with sensitivity to 1 drug. This gene plays a role in monocytic differentiation [36] and it is a monocytic AML marker [37]. *MARCH1*’s overexpression is correlated with sensitivity to 9 drugs and it promotes the proliferation of AML cells and inhibits apoptosis and differentiation [38]. *CYSLTR2*’s overexpression is correlated with sensitivity to 8 drugs and it is a receptor for the inflammatory mediators cysteinyl leukotrienes and overexpressed in *FLT3*-ITD- and *NPM1-*mutated AML samples [39]. PCBP3’s under expression is correlated with sensitivity to 8 drugs and its high expression is relatable to favorable survival [40].

### Consistency of kTSP rules across clinically distinct cohorts

To investigate the consistency and generalizability of the learned kTSP rules independent of predictive accuracy, we analyzed how the optimal rules, derived from models trained on the entire Beat AML dataset (waves *1–4*), applied to specific patient subgroups.

We defined a rule compliance metric as the sum of votes: a rule (*geneA>geneB*) applied to a sample contributed *+1* vote, and if not, it counted as *−1*. This sum reflects the degree to which the learned rule set maintains directional consistency within a subgroup.

We performed two key comparisons:

1. Temporal Consistency: We compared the rule compliance between the temporal training and testing cohorts (Waves *1 + 2* versus Waves *3 + 4*) (S3A Fig).
2. Disease Origin Consistency: We compared the rule compliance between de novo AML and not de novo AML (S3B Fig).

As S3 Fig demonstrates, there is high consistency of the kTSP rules across these different patient stratifications, validating the robustness of the relative expression signature.

## Discussion

In this study, we analyzed the predictive power of relative gene expression for drug response prediction in Acute Myeloid Leukemia (AML). Our approach utilized the kTSP classifier, training a robust model with a small, interpretable signature of no more than 15 gene pairs. Crucially, kTSP does not rely on actual gene expression measurements but only on the relative expression ordering (*geneA>geneB*), making it inherently robust to batch effects.

As demonstrated by the significant batch effect in our PCA (Fig 3), absolute expression values vary drastically across platforms. kTSP’s reliance on relative ordering bypasses this variance, explaining its superior generalizability to the FPMTB cohort.

The robustness of kTSP has significant clinical implications. It can help prioritize treatment for AML patients regardless of the expression data platform used, provided the relative expressions of the classifier gene pairs remain consistent. Even if a few gene pair orders change, the classifier remains useful because the final sensitivity call depends on the unweighted majority vote of all rules. This feature eliminates the need for complex batch correction or alignment of a test cohort to the training dataset, making it uniquely suited for single-patient classification. This enables real-time clinical decision-making the moment a single patient’s transcriptomic data is available. Furthermore, the kTSP classifier requires measuring only a small, fixed number of genes, making it compatible with high-throughput, cost-effective solutions like quantitative PCR (qPCR). This approach can replace ex vivo experiments in health facilities where it is not feasible to perform them. This is a significant practical advantage, as our results show it achieves this simplicity while maintaining accuracy comparable to or higher than complex classifiers like SVM, elastic net, and random forest.

### Novelty and interpretability

The kTSP classifier has previously been applied to tasks like tumor type classification [41] and prognosis prediction [25]. To the best of our knowledge, this study represents the first application of kTSP to *ex vivo* drug response prediction using patient-derived AML samples. Our completely data-driven approach proved robust to both imbalanced class sizes and batch effects. We anticipate that the classifier rules provided in Table in S1 Table for single drug and Table in S2 Table for venetoclax containing combination drugs can be readily adopted in clinics or research centers where *ex vivo* drug screening is infeasible, as prediction requires only checking the simple majority voting logic, eliminating the need for complex software tools.

### Limitations and future directions

A key limitation of this study is that the models were trained using patient-derived *ex vivo* drug responses, meaning the predictions may not always perfectly align with a patient’s in vivo (clinical) response to treatment. Another limitation stems from the binary classification: samples with intermediate drug responses are assigned to either the resistant or sensitive label. This discretization process of the AUC values (using the *AUC = 100* cutoff) may cause a loss of precision, although our cutoff aligns with the standards used in the Beat AML studies. Future work could address this by exploring the multiclass classification extension of the algorithm, multiclassPairs [42], which can classify samples as responsive, resistant, and intermediate. Future studies should also compare performance against thresholds optimized specifically for clinical endpoints.

## Conclusion

We believe this study provides a useful contribution to the literature, particularly for classification tasks characterized by imbalanced class distributions. We successfully validated our kTSP classifiers on an external dataset without performing any batch correction. For many drugs, including venetoclax and sorafenib, we observed that the sDSS scores were significantly higher in kTSP-classified sensitive samples compared to resistant samples. We specifically highlighted venetoclax and sorafenib as they represent distinct classes of targeted therapies—BCL-2 inhibitors and multi-kinase inhibitors, respectively—that are clinically pivotal in the treatment of hematologic malignancies. Venetoclax has revolutionized AML treatment for older patients who cannot handle standard chemotherapy. Sorafenib is frequently used for AML patients with specific mutations (like *FLT3-ITD*). Finally, we verified the strong consistency of the rules across both the temporal split of the Beat AML data (Waves *1 + 2*) vs. (Waves *3 + 4*) and clinically relevant disease origins (*de novo* vs. *not de novo*). The high consistency of rules across de novo and not de novo AML cases (S3 Fig) suggests that the underlying gene-pair relationships capture fundamental mechanisms of drug sensitivity that transcend the patient’s clinical history.

## Supporting information

S1 Fig (A) Area Under the Drug Response Curve (AUC) values for drugs in the Beat AML Waves 1 + 2 (training) and Waves 3 + 4 (testing) cohorts used for single-drug response prediction. (B) and for venetoclax containing combination drugs (C) Distribution of *ex vivo* drug response (AUC) for all samples in the Eide et *al.* cohort independent of being utilized in this manuscript.
(PDF)

S2 Fig The relationship between the datasets and analyses performed in this manuscript. (A) The overall schema (B) The flowchart of the analyses.
(PDF)

S3 Fig Consistency of rules among different patient groups. Sum of votes (y axes) is the sum of rules that apply/don’t apply, a rule (*geneA>geneB*) applied to a sample contributed *+1* vote, and if not, it counted as *−1*. This sum reflects the degree to which the learned rule set maintains directional consistency within a subgroup. (A) The sum of rules that apply/don’t apply for each Beat AML cohort (Waves 1 + 2 vs. Waves 3 + 4) (B) The sum of rules that apply/don’t apply for the patients that are de novo or not. We included the drugs that have at least 20 samples for each group shown.
(PDF)

S1 Table The classifier rules for single drugs. (XLSX)

S2 Table The classifier rules for venetoclax containing combination drugs. (XLSX)
