# Supplementary figures and images for: Robust predictors for drug response of patients with acute myeloid leukemia
Source: PLoS One. 2026 Feb 23;21(2):e0343422. doi: 10.1371/journal.pone.0343422 (PMC12928390; doi:10.1371/journal.pone.0343422)

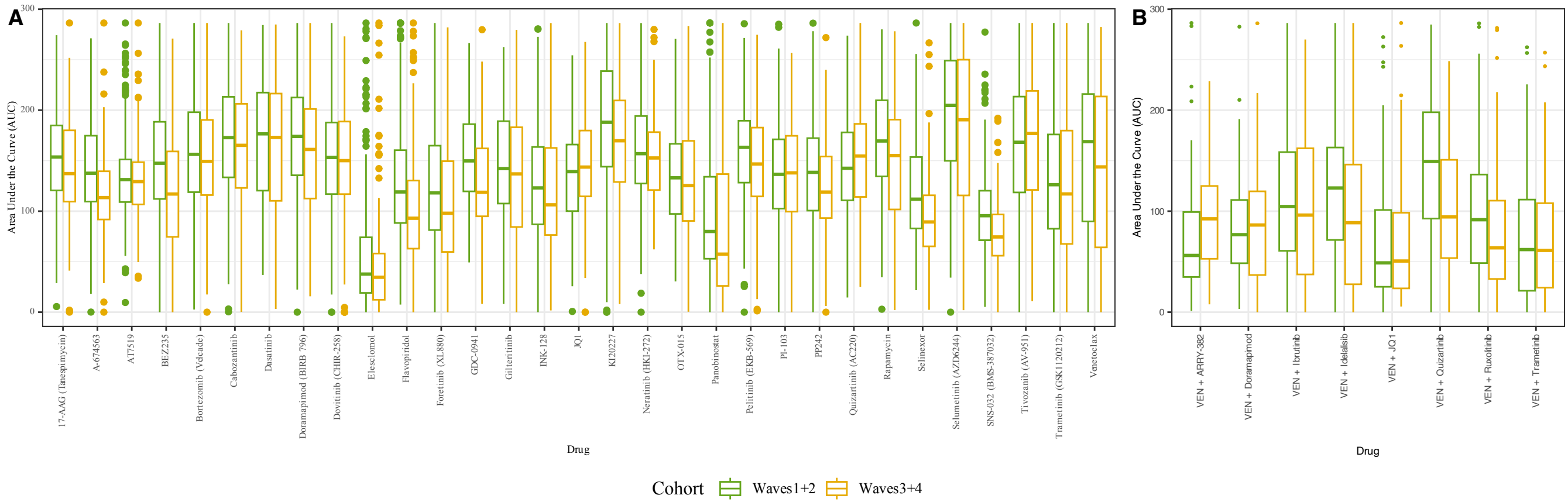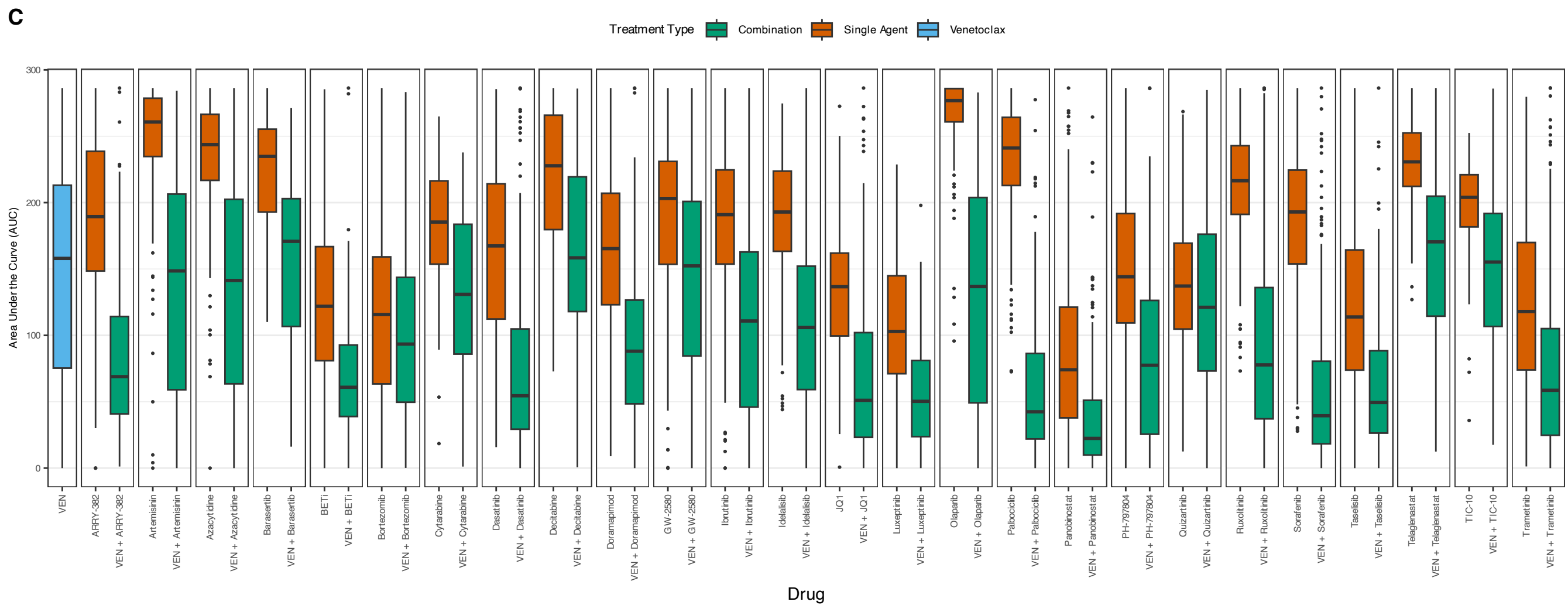

Supplement: S1 Fig — (B) and for venetoclax containing combination drugs (C) Distribution of ex vivo drug response (AUC) for all samples in the Eide et al. cohort independent of being utilized in this manuscript. (PDF) [file pone.0343422.s001.pdf]

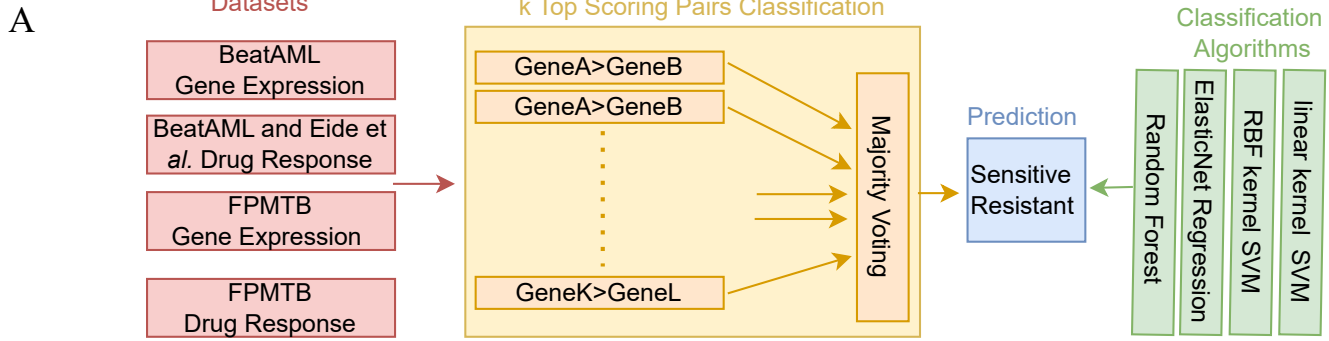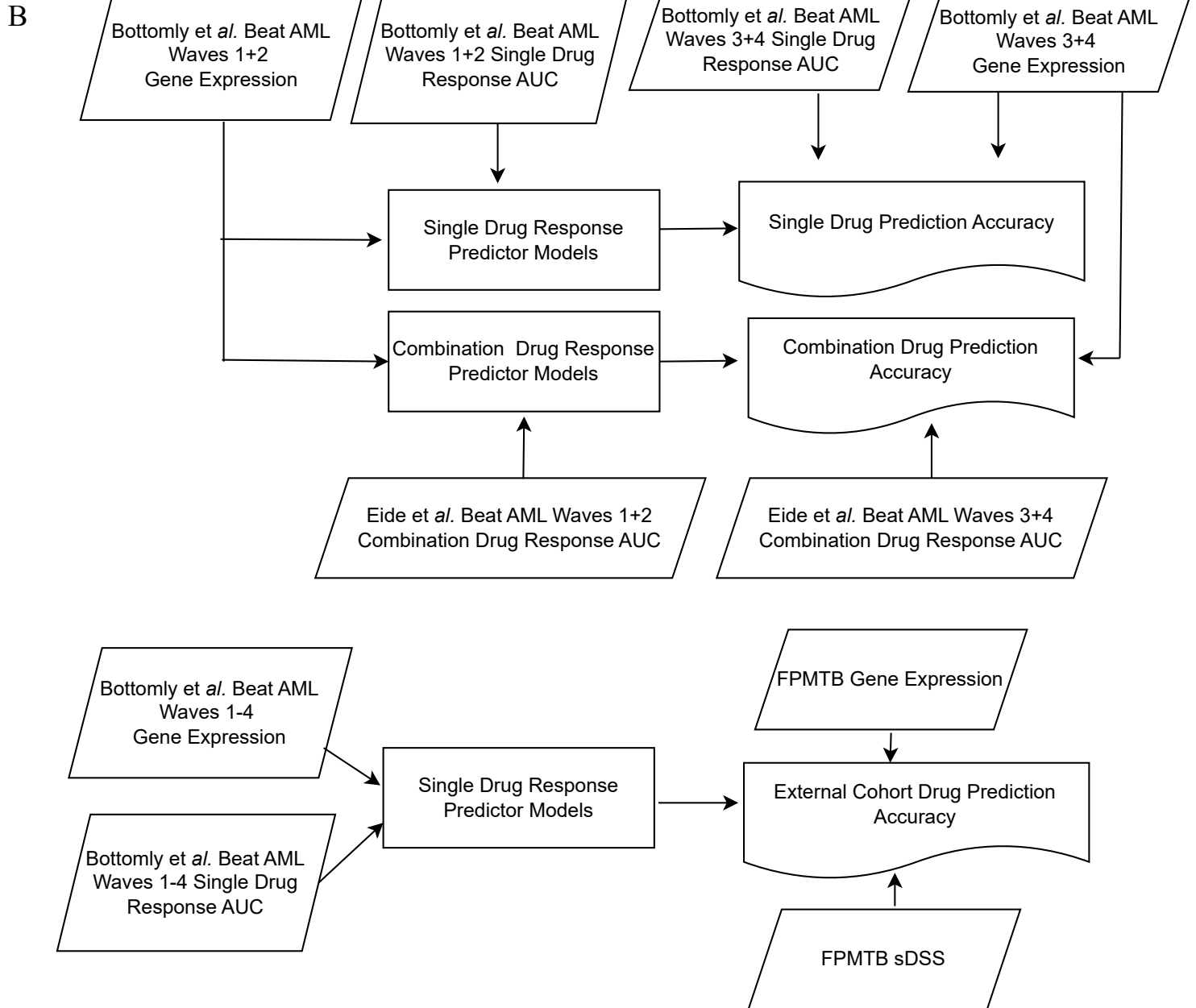

Supplement: S2 Fig — (A) The overall schema (B) The flowchart of the analyses. (PDF) [file pone.0343422.s002.pdf]

**A**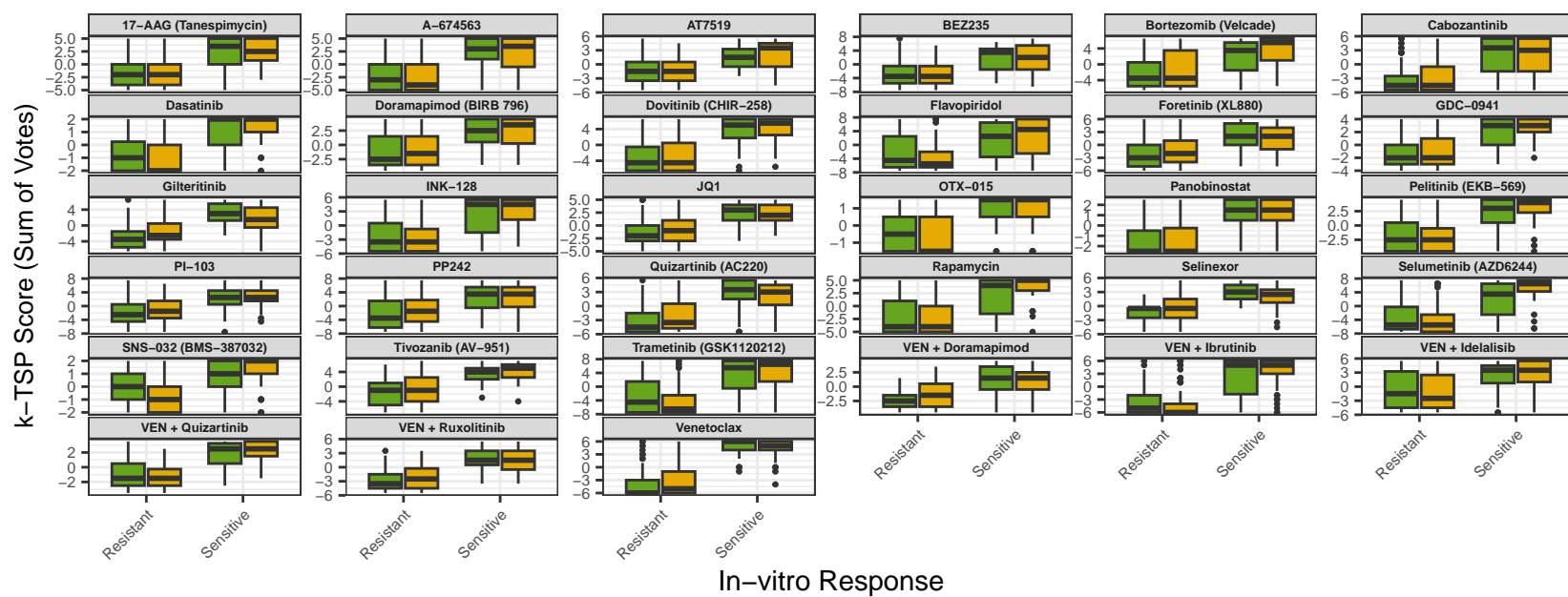**B**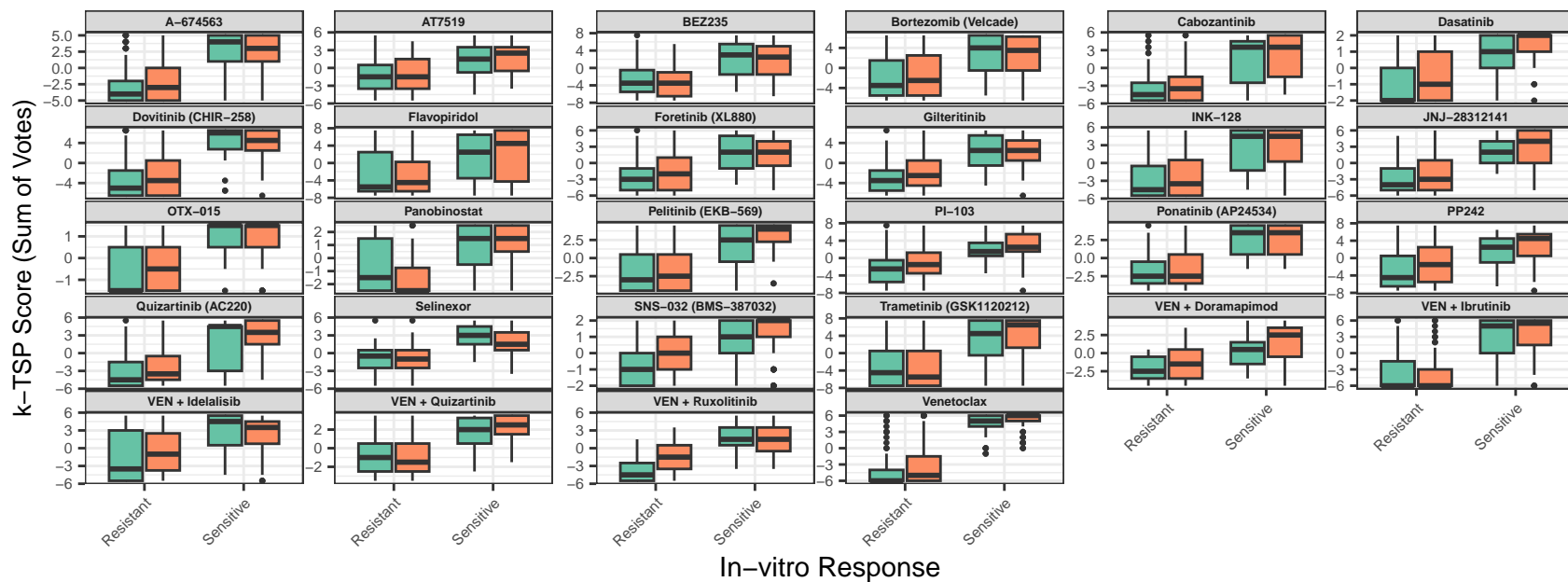

Supplement: S3 Fig — Sum of votes (y axes) is the sum of rules that apply/don’t apply, a rule (geneA>geneB) applied to a sample contributed +1 vote, and if not, it counted as −1. This sum reflects the degree to which the learned rule set maintains directional consistency within a subgroup. (A) The sum of rules that apply/don’t apply for each Beat AML cohort (Waves 1 + 2 vs. Waves 3 + 4) (B) The sum of rules that apply/don’t apply for the patients that are de novo or not. We included the drugs that have at least 20 samples for each group shown. (PDF) [file pone.0343422.s003.pdf]
